# In vitro and in vivo studies on anti-malarial activity of *Commiphora africana* and *Dichrostachys cinerea* used by the Maasai in Arusha region, Tanzania

**DOI:** 10.1186/s12936-019-2752-8

**Published:** 2019-04-04

**Authors:** Prisca A. Kweyamba, Denis Zofou, Noella Efange, Jules-Clement N. Assob, Jovin Kitau, Mramba Nyindo

**Affiliations:** 1 Department of Parasitology and Entomology, Kilimanjaro Christian Medical University College, P. O. Box 2240, Moshi, Tanzania; 2 Medical Research and Applied Biochemistry Laboratory (Drug Discovery and Development Research Unit), University of Buea, P.O. Box 63, Buea, South West Region Cameroon

**Keywords:** Phytochemical screening, *Commiphora africana*, *Dichrostachys cinerea*, Antimalarial and cytotoxicity

## Abstract

**Background:**

Traditional medicinal plants are one of the potential sources of anti-malarial drugs and there is an increasing interest in the use and development of traditional herbal remedies for the treatment of malaria and other ailments. This study was carried out with the aim to investigate the phytochemical screening, cytotoxic effect and antiplasmodial activities of *Dichrostachys cinerea* and *Commiphora africana*. Both plants are used by the Maasai in Tanzania in suspected malaria and other diseases. No previous work appears to have investigated the potential anti-malarial activity of the two plants.

**Methods:**

This study aimed to investigate the in vitro anti-malarial activity of methanol and dichloromethane extracts of the two plants against chloroquine sensitive (D6) and chloroquine resistant (Dd2) strains of *Plasmodium falciparum*. The anti-malarial property was assessed by the lactate dehydrogenase method (pLDH). The in vivo anti-malarial study was carried out using the Peters’ 4-day suppressive test in *Plasmodium berghei* in Balb/c mice. Cytotoxic tests were carried out using monkey kidney epithelial cell line in [3(4,5-dimethylthiazol-2-yl)-2,5-diphenyltetrazolium bromide] (MTT) assay. Qualitative phytochemical screening was carried out using standard methods of analysis.

**Results:**

The phytochemical screening of plant extracts revealed the presence of alkaloids, flavonoids, tannins, steroids, triterpenoids, glycosides and saponins. However, alkaloids were absent in most plant extracts. The dichloromethane extracts of *C. africana* (stem bark); *D. cinerea* (stem bark) and methanol extracts of *D. cinerea* (whole stem) all showed promising in vitro anti-malarial activities. All other extracts did not show any significant anti-malarial activity. The two most promising extracts based on in vitro studies, DCM extracts of *C. africana* (stem bark) and *D. cinerea* (stems bark), equally exhibited very significant anti-malarial activities in the mouse model. They exhibited parasite suppression rates of 64.24 and 53.12%, respectively, and considerable improvement in weight and survival rate. Most plant extracts were not cytotoxic except for DCM extract of *D. cinerea* (whole stem) CC_50_ (29.44 µg/mL).

**Conclusion:**

The findings of this study provide scientific evidence supporting the traditional use of the plants in the treatment of malaria by the Maasai in Arusha region, Tanzania. Consequently, further work including bioassay-guided fractionation and advanced toxicity testing may yield new anti-malarial drug candidates from the two plants.

## Background

Malaria is a potentially fatal tropical disease that is caused by a Protozoa of the genus *Plasmodium*. Of species of the parasites that affect humans, *Plasmodium falciparum* is by far the most virulent. The 2017 World Health Organization (WHO) Malaria report estimated 216 million cases of malaria and 445,000 deaths worldwide, with 90% of cases and 91% of deaths affecting the WHO African region [1, 2]. Tanzania has entered a new era of malaria control with a realistic possibility to reduce malaria prevalence to less than 1% by 2020 through the national malaria strategy plan [3]. However, the prevalence of malaria in Tanzania has risen from 9% in 2011–2012 to 14% in 2015–2016, with the highest risk being areas close to the Lake zone and in rural settings [4].

The battle against malaria is becoming a serious challenge due to emergence of *P. falciparum* strains resistant to all anti-malarial drugs, including artemisinin-based combinations [2]. Therefore, there is a need to continuously identify new chemical compounds with anti-malarial activity. For decades, medicinal plants have played an important role in malaria treatment with the discovery of two major drugs, quinine and artemisinin that are used worldwide. Thus, medicinal plants have a great potential to provide new anti-malarial substances. It is also estimated that 80% of the population in developing countries depend on medicinal plants to meet their primary health care needs [5].

In Tanzania, scientific investigations have shown that over 100 plant species are used mainly by the rural population for the treatment of malaria, whereby some have been reported to have significant anti-malarial activity, but not many have been documented and only a few have been tested for anti-malarial activity [6]. The present study aimed at screening crude extracts from two widely used plants by the Maasai namely, *Commiphora africana* (Family Burseraceae) and *Dichrostachys cinerea* (Family Fabaceae) for their traditional use as anti-malarial.

## Methods

### Collection of plant materials

Stem bark and whole stem of both *D. cinerea* and *C. africana* were collected in Monduli district, Arusha region in Tanzania in October 2017. The botanic identification of the specimens was confirmed by a qualified botanist at the Tanzania National Herbarium in Arusha, where vouchers No PAK 001 and PAK 002 were deposited.

### Plant extraction

For each plant sample, 60 g of dried ground plant material was repeatedly extracted in 1000 mL of dichloromethane and methanol in a 1:1 ratio (v/v). Plant extracts were filtered with Whatman Grade 1 filter paper, and the solvent was concentrated to dryness under reduced pressure using a BÜCHI R-200 rotavapor. The dry extracts were weighed, transferred into vials and stored at − 20 °C for further analysis.

### Phytochemical analysis

Preliminary phytochemical analysis of plant extracts was performed to screen the plants for selected phytoconstituents including; alkaloids, steroids, cardiac glycosides, flavonoids, tannins and saponins using standard methods with some modifications [7–10].

### *Plasmodium falciparum *culture and maintenance

A continuous culture of *P. falciparum* malaria parasites was established using the method described by Trager and Jensen with some modifications [11, 12]. The *Plasmodium* strains used were: D6 (chloroquine sensitive strain) and Dd2 (chloroquine resistant strain), isolated from Sierra Leone and Thailand, respectively. Parasites were grown in uninfected O+ RBCs as host cells and maintained in RPMI-1640 medium which was supplemented with 2 mg/mL NaHCO_3_, 10 µg/mL hypoxanthine, 2 mg/mL glucose, 1% albumax II and 10 µg/mL gentamicin. The culture was maintained at 37 °C in a CO_2_ incubator. Parasitaemia was determined qualitatively and quantitatively using a fluorescent microscope (DAPI) and light microscopy (Giemsa stain).

### In vitro antiplasmodial activity

In vitro antiplasmodial activity of the plant extracts was done in 96 well plates. Wells with parasitized red blood cells and without plant extract served as negative controls whereas wells containing cultures with chloroquine diphosphate served as positive controls. The plates containing parasite cultures were incubated for 48 h at 37 °C in a CO_2_ incubator. After 48 h of incubation, the plates were frozen overnight at − 20 °C and antiplasmodial activity was assessed using the parasite lactate dehydrogenase (pLDH) assay that was previously described [12, 13]. The pLDH assay generated optical density values at various concentrations of the plant extracts using the Graphpad prism version 6 software (https://www.graphpad.com/scientific-software.html). A log dose response curve was generated and used to determine IC_50_ and CC_50_. Each product was tested in duplicate and the IC_50_ and CC_50_ values obtained from the duplicates were pooled and expressed as geometric means and standard deviations. The independent sample t-test was used to compare mean IC_50_ of anti-malarial activity between plant extracts using STATA version 13 (software Stata-Corp, College Station, TX, USA).

### Cytotoxicity

Cytotoxicity of each plant extracts was assessed on LLC-MK2 monkey kidney epithelial cells. The cells were cultured in DMEM medium which was supplemented with 10% fetal bovine serum and 10 mg/mL of penicillin–streptomycin. The cells were then seeded in 96 well plates at 10,000 cells per well in 100 µL culture medium. The cells were incubated for 48 h at 37 °C in a CO_2_ incubator until they reached a level of confluence. After 48 h, the medium was completely removed from the wells by inverting the microtiter plate and by tapping it on a sterile filter paper. The medium was then replaced with 100 µL of fresh culture medium, followed by 100 µL of crude plant extract (1000 µg/mL) in row H of the 96 well plate and serially diluted twofold to give concentrations ranging from 500 to 7.8125 µg/mL. Row A of the 96 well plate served as control wells, whereby the negative control contained culture medium and LLC-MK2 cells without plant extract (100% growth). The cells were maintained at 37 °C in a CO_2_ incubator before determining their viability by using the MTT assay as previously described [6].

### In vivo pharmacological studies

#### Experimental animals and parasite

Balb/c albino mice (Male and Female) aged 6–8 weeks and weighing 15–32 g used for this study, were provided by the Animal Breeding Unit of the Medical Research and Applied Biochemistry Laboratory (Drug Discovery and Development Research Unit) at the University of Buea. They were maintained at a room temperature of about 25 °C and 12:12 light/dark cycle, with food and water given ad libitum*.* All experiments were conducted with regards to the internationally accepted laboratory animal use, care and guidelines. The project proposal and SOPs were reviewed and approved by the University of Buea Institutional Ethics Review Board for Animal Use.

The lethal strain of *Plasmodium berghei* was kindly donated by BEI-Resources, Manassas, USA. To maintain the parasite, weekly serial passage of blood from infected mice to non-infected mice were performed [14].

#### Parasite inoculation

Donor albino mice previously infected with *P. berghei* and having parasitaemia level of 20–30% were used. The donor mice were anesthetized and sacrificed by opening the thoracic region in order to expose the heart. Blood was collected by cardiac puncture into heparinized vacutainer tube containing 0.5% trisodium citrate. Physiological saline (0.9%) was used to dilute the blood based on parasitaemia level of the donor mice [15].

### In vivo suppressive test of *Plasmodium berghei*

The 4-day suppressive test was used to measure the schizonticidal activity of the bioactive extracts and controls against *P. berghei* infected Balb/c mice, following a method described by Peters et al*. *[16], with some modifications [6]. In brief, infected mice were randomly divided into four groups of 5 each by weight. Treatment started 3 h after mice had been inoculated with the parasite on day 1 and then continued daily for 4 days (that is from day 1 to day 5). For each extract, animals received daily oral dose of 400 mg/kg day in 100 µL phosphate buffer saline (PBS), pH 7.4. The positive control-group received 10 mg/kg body weight quinine orally per day, while the negative-control group animals were administered 100 µL of PBS. The survival rate was monitored on a daily basis in all groups for 28 days post-inoculation. On day 5, Giemsa-stained thin blood smears were prepared from the tail of each animal to determine parasitaemia and percentage inhibition [17]. The percentage of parasite growth suppression (PGS) was estimated using the following equation:

$$ {\text{PGS }} =  100  \times  \left[ {\left( {A - B} \right)/A} \right], $$

where *A* is the average parasitaemia of the negative-control group and *B* corresponds to the parasitaemia of the test group. The mean survival time for each group was determined in each group over a period of 28 days.

## Results

### Preliminary phytochemical screening

Preliminary phytochemical screening results of *D. cinerea* and *C. africana* extracts which were tested for eight selected phytoconstituents are summarized in Table 1. MeOH extracts of both plants showed presence of cardiac glycosides, flavonoids, tannins, triterpenoids and saponins. However, presence of alkaloids was only observed in *C. africana* extracts. Steroids were absent in all MeOH extracts. DCM extracts had appreciable levels of steroids, saponins and flavonoids. Surprisingly, these extracts did not contain any tannins, triterpenoids and alkaloids.

**Table 1:** Preliminary phytochemical results for *Dichrostachys cinerea *and *Commiphora africana*

Phytochemical constituents	DCM extracts				MeOH extracts			
	CAS	CAB	DCS	DCB	CAS	CAB	DCS	DCB
Alkaloids	−	−	−	−	++	+	−	−
Steroids	+ ++	+++	++	+	−	−	−	−
Cardiac glycosides	+	−	−	−	+++	+++	+++	+++
Flavonoids	+++	+++	+++	+++	+++	+++	+++	+++
Tannins	−	−	−	−	+++	+++	+++	−
Triterpenoids	−	−	−	−	+++	++	+++	++
Saponins	+++	+++	+++	+++	+++	++	+	+++

CAS: *Commiphora africana* whole stem; CAB: *Commiphora africana* stem bark; DCS: *Dichrostachys cinerea* whole stem; DCB: *Dichrostachys cinerea* stem bark; (+++): strongly present; (++): moderately present; (+): weakly present; (−): absent

### In vitro antiplasmodial activity and cytotoxicity of plant extracts from *Dichrostachys cinerea *and *Commiphora africana*

Table 2 shows the in vitro antiplasmodial activity of all plant extracts prepared from the selected plants, as well as their cytotoxic effect on LLC-MK2 cells. Of the eight extracts tested, DCM extracts of *C. africana* stem bark and *D. cinerea* whole stem showed promising antiplasmodial activity (IC_50_ = 4.54 ± 1.80 µg/mL and 11.47 ± 2.17 µg/mL, respectively) against D6 strain of *P. falciparum.* However, DCM extract of *D. cinerea* stem bark showed promising activity against both D6 and Dd2 strains of *P. falciparum* (IC_50_ = 2.37 ± 0.86 µg/mL and 11.92 ± 7.43µg/mL, respectively). The only methanolic extract with promising activity was that of *D. cinerea* whole stem (IC_50_ = 2.96 ± 1.87 µg/mL) against the D6 strain. According to the cytotoxic classification [16] all plant extracts were reported to be non-toxic to LLC-MK2 cells with CC_50_ of > 30 µg/mL except for DCM extract of *D. cinerea* whole stem which was reported to be weakly toxic (CC_50_ = 29.44 ± 4.16 µg/mL).

**Table 2:** In vitro antiplasmodial activity and cytotoxicity of crude extracts from *Dichrostachys cinerea *and *Commiphora africana*

Extract	IC_50_ on D6 (µg/mL ± SD)	IC_50_ on Dd2 (µg/mL ± SD)	CC_50_ on LLC-MK2 (µg/mL ± SD)	Selectivity index by D6
CAB-DCM	*4.54 ± 1.80*	> 1000	> 1000	*471.00*
CAS-DCM	> 1000	> 1000	> 1000	ND
CAB-MeOH	> 1000	> 1000	> 1000	ND
CAS-MeOH	> 1000	> 1000	74.56	ND
DCB-DCM	*2.37 ± 0.86*	*11.92 ± 7.43*	34.65 ± 8.36	14.63
DCS-DCM	*11.47 ± 2.17*	> 1000	*29.44 ± 4.16*	*2.57*
DCB-MeOH	> 1000	> 1000	> 1000	ND
DCS-MeOH	*2.96 ± 1.87*	> 1000	178.35 ± 371.30	60.24
QN	*0.05 ± 0.01*	*0.06 ± 0.01*		
Triton X-100			*2.44 ± 0.82*	

Italic values represent anti-plasmodial activity

Plant part: CAS: *Commiphora africana* whole stem; CAB: *Commiphora africana* stem bark; DCS: *Dichrostachys cinerea* whole stem; DCB: *Dichrostachys cinerea* stem bark

QN: quinine; ND: not determined; SD: standard deviation

Findings from the in vivo anti-malarial study are summarized in Table 3. These results show that animals treated with the extracts had significantly higher survival rates during the 28 days of observation as compare to the control group, though lower than the one in which quinine was used. These groups also experienced a steady constant increase in body weight, and parasite suppression rates above 50%.

**Table 3:** In vivo anti-malarial activities of crude extract of *Dichrostachys cinerea *and *Commiphora africana *in the Peters’ suppressive test

Treatment	Parasite suppression rate (%)	Change in body weight (initial–final in grams)	Mean survival days
Control	0.00	− 17.9% (23.5 ± 1.1–19.3.0 ± 1.4)	12.2 ± 2.5
CAB-DCM	64.24 ± 1.43	5.8% (22.4 ± 0.6–23.7 ± 1.2)	23.0 ± 3.5
DCB-DCM	53.12 ± 2.02	3.9% (22.1 ± 1.0–22.9 ± 1.1)	22.3 ± 3.05
Quinine	96.02 ± 5.00	7.2% (20.8 ± 1.5–22.3 ± 1.0)	28.0 ± 0.0

Mean and standard deviation values were generated from five replicates of each assay

CAB-DCM: dichloromethane extract of *Commiphora africana* bark; DCB-DCM: dichloromethane extract of *Dichrostachys cinerea* bark

## Discussion

Plant extracts of *D. cinerea* and *C. africana* were screened for presence of phytochemicals which were then tested for anti-malarial activity against *P. falciparum* strains and cytotoxicity on LLC-MK2 monkey kidney epithelial cells. The methanol solvent showed high levels of phytoconstituents extracted, with strong presence of polar phytoconstituents such as tannins, cardiac glycosides and flavonoids. Dhawan and Gupta also reported that methanol as an extraction solvent worked best for the extraction of various active phytochemicals [18].

From the studied medicinal plants extracts, *C. africana* showed a higher diversity of phytoconstituents than *D. cinerea*. Flavonoids and saponins were strongly present in both plants and these findings are consistent with similar studies conducted in Zimbabwe and Burkina Faso [19, 20]. Alkaloids on the other hand, were weakly or moderately present only in the *C. africana* plant. A plausible explanation for this could be because alkaloids are known to exist in large proportions in the seeds and roots [21], whereas in this study the plant parts used were the stem bark and whole stem. Additionally, some phytochemicals are known to possess various pharmacological effects and may be responsible for various actions in *D. cinerea* and *C. africana*. For example, previous studies have reported that flavonoids possess notable antioxidant activity [22] and saponins having anti-inflammatory activities [23].

There are several strategies that are available for the discovery of new anti-malarial drugs and therefore identification of traditionally used plants extracts exhibiting IC_50_ values of less than 15 µg/mL are important first steps in the search for new anti-malarial plant extracts. In our study *D. cinerea* extracts exhibited high in vitro antiplasmodial activity, with the DCM stem bark extract having promising activity (2.37 ± 0.86 and 11.92 ± 7.43 µg/mL, respectively) against both CQ sensitive (D6) and CQ resistant (Dd2) strains and was also found to be non-cytotoxic to the LLC-MK2 cells 34.65 ± 8.36 μg/mL. Therefore, the findings against CQ resistant *P. falciparum* parasites and non-toxic results to LLC-MK2 cells suggest that the extract can be a potential source for the isolation of safe and effective anti-malarial lead compounds. Similar studies conducted in South Africa, also reported *D. cinerea* to have promising antiplasmodial activity of 2.10 µg/mL against NF54 of *P. falciparum* [24].

The DCM extract of *D. cinerea* whole stem (DCS-MeOH) had promising antiplasmodial activity (11.47 ± 2.17 µg/mL), however it was found to be weakly cytotoxic (29.44 ± 4.16 µg/mL) and a selectivity index of 2.57, which suggests that the promising anti-malarial activity noticed was probably due to cytotoxicity rate than activity against the *P. falciparum* parasites per se.

The methanol crude extract of *D. cinerea* whole stem also had promising antiplasmodial activity (2.96 ± 1.87 µg/mL) and this plant extract contained the majority of phytoconstituents selected. Perhaps, the synergism among the phytoconstituents that comprise the extract could explain the in vitro antiplasmodial activity. Surprisingly, most of the *C. africana* extracts were inactive despite their traditional use as anti-malarials by the Maasai. Its low activity could be due to variation in the active constituents possibly related to seasonal or geographical setting and also ability of phytoconstituents to mask the activity of other phytoconstituents which may result in reduction of the activity of the extract [25]. However, the DCM extract of the bark exhibited the highest effectiveness in *P. berghei*-infected mice, with up to 64.24% parasite suppression rate, as compared to 53.12% observed with the DCM extract of the *D. cinerea* stem bark. The decline in parasite growth and/or multiplication also reflected as significant weight recovery and survival rate, as compared to the control group. These activities demonstrated in the animal model therefore reinforce the likelihood of these two plants to serve as important sources of new anti-malarial therapies. Further investigations are urgently envisaged, as there is no published work on anti-malarial and cytotoxicity activity of *C. africana* so far.

## Conclusion

In conclusion, this study has shown the pharmacological basis of the two plants that the Maasai in Arusha region in Tanzania use to treat suspected cases of malaria. In particular the antiplasmodial activity of dichloromethane, stem bark extract of *D. cinerea* showed good in vitro activity against the *P. falciparum* chloroquine resistant strain. The ability of this preparation to kill chloroquine resistant parasite strains is of value where chloroquine resistant malaria is in existence. Also there was a significant parasite suppression and disease recovery ability in *P. berghei* animal model. In addition, methanol extracts of *D. cinerea* (DCS-MeOH) had a cytotoxic concentration (CC_50_) of 178.35 µg/mL, suggesting that the plant extracts can be used to develop usable anti-malarials. Consequently, further work including bioassay-guided fractionation, and toxicity testing are recommended that can yield new anti-malarial drug candidates.

## Abbreviations

DCM: dichloromethane
DMEM: Dulbecco’s Modified Eagle’s medium
CC_50_: 50% cytotoxic concentration
IC_50_: 50% inhibitory concentration
MeOH: methanol
RPMI: Roswell Park Memorial Institute
